# Clinical factors underlying a single surgery or repetitive surgeries to treat superior oblique muscle palsy

**DOI:** 10.1186/s40064-015-0945-3

**Published:** 2015-04-07

**Authors:** Kana Aoba, Toshihiko Matsuo, Ichiro Hamasaki, Kayoko Hasebe

**Affiliations:** Department of Ophthalmology, Okayama University Medical School and Graduate School of Medicine, Dentistry, and Pharmaceutical Sciences, Okayama, Japan; Present address: Department of Ophthalmology, Kawasaki Medical School-Affiliated Kawasaki Hospital, Okayama, Japan

**Keywords:** Superior oblique muscle palsy, Surgery, Inferior oblique muscle recession, Inferior rectus muscle recession, Vertical deviation, Cyclotorsional deviation (torsion), 95% confidence interval, Informed consent, Repetitive surgeries, Superior rectus muscle recession

## Abstract

The purpose of this study is to know clinical factors underlying either a single surgery or repetitive surgeries, required to treat superior oblique muscle palsy. Retrospective review was made on 246 consecutive patients with idiopathic (n = 212) or acquired (n = 34) superior oblique muscle palsy who underwent surgeries in 8 years at one institution. Idiopathic palsy included congenital and decompensated palsies while acquired palsy included traumatic and ischemic palsies. Clinical factors, compared between groups with a single surgery (n = 203) and two or more surgeries (n = 43), were surgical methods, sex, age at surgery, horizontal, vertical, and cyclotorsional deviations, and stereopsis at near fixation. Inferior oblique muscle recession on paretic side was chosen in about 60% of the single-surgery and repetitive-surgery group as an initial surgery, followed by inferior rectus muscle recession on non-paretic side. The age at surgery was significantly older, vertical and cyclotorsional deviations were significantly larger in the repetitive-surgery group, compared with the single-surgery group (*P* = 0.01, *P* < 0.001, *P* = 0.02, Mann–Whitney U-test, respectively). The 95% confidence interval of vertical deviations was 15–17 prism diopters in the single-surgery group and 23–28 prism diopters in the repetitive surgery group. Significant differences in vertical deviations were replicated also in subgroups of patients with either idiopathic or acquired palsy. In conclusions, the 95% confidence interval of vertical deviations, determined by alternate prism and cover test, would be used as a common benchmark for predicting either a single surgery or repetitive surgeries, required to treat idiopathic and acquired superior oblique muscle palsy, in the process of obtaining the informed consent.

## Background

Superior oblique muscle palsy is a most frequent ocular motor abnormality, encountered in ophthalmic practice. The palsy is presented as either congenital or acquired. Congenital superior oblique muscle palsy is found in childhood usually with abnormal head posture, compensatory head tilt to the contralateral non-paretic side. Furthermore, the congenital palsy is sometimes diagnosed only in adulthood as decompensated palsy when diplopia and asthenopia cannot be erased by abnormal head posture and become manifest. The conditions are altogether designated as idiopathic palsy. The idiopathic superior oblique muscle palsy has genetic background, as evidenced by the familial occurrence (Astle and Rosenbaum 1985; Harris et al. 1986; Botelho and Giangiacomo 1996; Bhola et al. 2001; Imai et al. 2008), and by the muscle hypoplasia or the absence (Sawa 1978; Matsuo et al. 1988; Helveston et al. 1992; Plager 1992; Demer and Miller 1995; Sato 1999; Chan and Demer 1999; Sato et al. 2008; Uchiyama et al. 2010). Single nucleotide polymorphisms (SNPs) of the genes, expressed in the brain stem trochlear nucleus, were also detected (Imai et al. 2008; Jiang et al. 2004, 2005; Ohkubo et al. 2012).

Acquired superior oblique muscle palsy is indeed the acquired trochlear nerve palsy which develops abruptly with blunt head trauma or vascular ischemic accidents. The trochlear nerve palsy has a high chance for spontaneous recovery usually in one to three months. Surgery is, therefore, scheduled at least after half a year when diplopia still remains. The acquired superior oblique muscle palsy presents clinical manifestations such as diplopia, especially in the downward gaze, and a smaller range of vertical fusion amplitudes, which are different from the manifestations in the idiopathic palsy.

Surgical intervention in idiopathic or acquired superior oblique muscle palsy is not necessarily applied to all cases of the palsy. The clinical manifestations, especially diplopia or the large extent of abnormal head posture, lead to surgical intervention. Even among patients with idiopathic or acquired palsies who undergo surgeries, the clinical presentations, such as vertical deviations and vertical fusion amplitudes, vary largely from patient to patient. A large range of vertical fusion amplitudes in the idiopathic palsy, varying from patient to patient, will give unpredictable surgical outcome while a small range of vertical fusion amplitudes in the acquired palsy leads to the need of more precise surgery. Ideally, the surgery should be finished in a single secession, but an additional surgery will be sometimes required. The combination of two surgical procedures at a single secession is a recommended choice, but is difficult to be followed by non-subspecialty ophthalmologists.

Notwithstanding the different clinical manifestations in idiopathic and acquired superior oblique muscle palsy, one common surgical strategy would be to plan a second secession of an additional surgery when the initial surgery is insufficient to correct the deviation. At an institution with the strategy to plan a second surgery, informed consent would be better obtained from the viewpoint of preoperative prediction, as to whether a single surgery or repetitive surgeries are expected to treat the superior oblique muscle palsy in a patient. To give an answer to this question, we conducted a retrospective study to find clinical factors which underlay a single surgery or repetitive surgeries, required to treat 246 consecutive patients with idiopathic (n = 212) or acquired (n = 34) superior oblique muscle palsy at one institution.

## Results

In overall 246 patients with either idiopathic or acquired superior oblique muscle palsy, inferior oblique muscle recession on the paretic side was chosen in about 60% of both the single-surgery group (n = 203) and the repetitive-surgery group (n = 43) as an initial surgery (Table 1), followed by inferior rectus muscle recession on the non-paretic side in patients with paretic eye fixation. The choice of surgical procedures at an initial surgery showed the same trend even when the patients were divided into subgroups with either idiopathic palsy (n = 212) or acquired palsy (n = 34). In the repetitive-surgery group (n = 43), the common surgical procedures at the second surgery were inferior rectus muscle recession on the non-paretic side or superior rectus muscle recession on the paretic side (Table 2).

**Table 1:** **Surgical procedures for superior oblique muscle palsy in the single-surgery group and the repetitive-surgery group at initial surgery**

	**Diagnosis**	**Fixation eye**		**IO recession in paretic eye**	**SR recession in paretic eye**	**IR recession in contralateral eye**	**SO advancement in paretic eye**	**SO advancement in both eyes**
Single surgery	Idiopathic	Contralateral eye	n = 103	83 (80.6%)	11 (10.7%)	8 (7.8%)	0 (0%)	1 (0.9%)
				6 with LR rec.	1 with LR rec.	1 with LR rec.		
				1 with IR rec.				
		Paretic eye	n = 72	31 (43.1%)	5 (6.9%)	35 (48.6%)	1 (1.4%)	0 (0%)
				2 with IR rec.		5 with LR rec.		
						1 with MR rec.		
	Acquired	Contralateral eye	n = 17	7 (41.2%)	2 (11.8%)	3 (17.6%)	2 (11.8%)	3 (17.6%)
				1 with LR rec.		1 with MR rec.		
		Paretic eye	n = 11	3 (27.3%)	1 (9.1%)	3 (27.3%)	2 (18.2%)	2 (18.2%)
Repetitive surgeries	Idiopathic	Contralateral eye	n = 20	17 (84.2%)	0 (0%)	3 (15.8%)	0 (0%)	0 (0%)
				2 with LR rec.		1 with SR rec.		
				2 with IR rec.				
				1 with SR rec.				
		Paretic eye	n = 17	10 (58.8%)	0 (0%)	7 (41.2%)	0 (0%)	0 (0%)
						1 with LR rec.		
	Acquired	Contralateral eye	n = 4	2 (50.0%)	1 (25.0%)	1 (25.0%)	0 (0%)	0 (0%)
		Paretic eye	n = 2	1 (50.0%)	1 (50.0%)	0 (0%)	0 (0%)	0 (0%)

In 10 patients with bilateral palsy, including 2 with idiopathic palsy and 8 with acquired palsy, the paretic eye is assigned to the eye with the more marked palsy.

IO, inferior oblique muscle; SR, superior rectus muscle; IR, inferior rectus muscle; SO, superior oblique muscle; LR, lateral rectus muscle; MR, medial rectus muscle; rec., recession.

**Table 2:** **Surgical procedures at second surgery in the repetitive-surgery group with superior oblique muscle palsy**

**Diagnosis**	**Fixation eye**		**IO recession in paretic eye**	**SR recession in paretic eye**	**IR recession in contralateral eye**	**IR advancement in contralateral eye**	**LR rec. & MR res. in contralateral eye**	**MR recession in paretic eye**
Idiopathic	Contralateral eye	n = 20	1 (5.0%)	3 (15.0%)	14 (70.0%)	2 (10.0%)	0 (0%)	0 (0%)
				1 with LR rec.	1 with SR rec.			
				1 with MR rec.				
	Paretic eye	n = 17	1 (5.9%)	3 (17.6%)	11 (64.7%)	1 (5.9%)	1 (5.9%)	0 (0%)
					1 with LR rec.			
Acquired	Contralateral eye	n = 4	0 (0%)	1 (25.0%)	1 (25.0%)	1 (25.0%)	0 (0%)	1 (25.0%)
	Paretic eye	n = 2	1 (50.0%)	1 (50.0%)	0 (0%)	0 (0%)	0 (0%)	0 (0%)

In one patient with idiopathic bilateral palsy, the paretic eye is assigned to the eye with the more marked palsy.

IO, inferior oblique muscle; SR, superior rectus muscle; IR, inferior rectus muscle; LR, lateral rectus muscle; MR, medial rectus muscle; rec., recession; res., resection.

Surgical procedures at third surgery in 7 patients are: IO recession in paretic eye (n = 1), SR recession in paretic eye (n = 2, 1 with LR rec.), SR advancement in paretic eye (n = 1), IR advancement in contralateral eye (n = 1), IR recession in paretic eye (n = 1), and MR recession in paretic eye (n = 1). Of these 7 patients, one patient underwent SR recession in paretic eye at forth surgery.

In the overall analysis, including both idiopathic and acquired superior oblique muscle palsy, the age at surgery was significantly older, vertical and cyclotorsional deviations were significantly larger in the repetitive-surgery group, compared with the single-surgery group (*P* = 0.01, *P* < 0.001, *P* = 0.02, Mann–Whitney U-test, respectively, Table 3). The other factors, the sex, diagnostic entities, horizontal deviations, stereoacuity at near fixation, the dominant eye, or the presence of dissociated vertical deviation, did not show significant differences between the single-surgery group and the repetitive-surgery group (Table 3). The successful alignment for vertical and horizontal deviations was obtained in both the single-surgery group and the repetitive-surgery group around one month after the final surgery (Table 3). The residual vertical deviations after the final surgery were significantly larger in the repetitive-surgery group than in the single-surgery group (*P* = 0.04, Mann–Whitney U-test).

**Table 3:** **Clinical factors in the single-surgery group and repetitive-surgery group of superior oblique muscle palsy including both idiopathic and acquired palsy**

		**Single surgery**	**Repetitive surgeries**	***P*** **value**	**Statistical method**
		**(n = 203)**	**(n = 43)**		
Sex					
Male		107 (52.7%)	19 (44.2%)	0.31	Fisher exact probability test
Female		96 (47.3%)	24 (55.8%)		
Diagnosis					
Idiopathic		175 (86.2%)	37 (86.0%)	0.98	Fisher exact probability test (idiopathic versus acquired)
Congenital		78	12		
Decompensated		97	25		
Acquired		28 (13.8%)	6 (14.0%)		
Traumatic		26	5		
Ischemic		2	1		
Age at surgery	Mean ± Standard deviation	40.5 ± 25.9	51.0 ± 20.9		
(year)	Minimum, Maximum (Median)	3.4, 83.0 (44.8)	5.3, 82.8 (53.8)	0.01	Mann–Whitney U-test
	95% Confidence interval	36.9 to 44.1	44.5 to 57.4		
Horizontal deviation	Mean ± Standard deviation	−4.3 ± 8.3	−7.3 ± 14.1		
(prism diopter)	Minimum, Maximum (Median)	−35, 20 (−2)	−41, 17 (−4)	0.31	Mann–Whitney U-test
-: exodeviation	95% Confidence interval	−3.2 to −5.5	−2.9 to −11.6		
+: esodeviation					
Vertical deviation	Mean ± Standard deviation	16.3 ± 8.1	26.1 ± 8.8		
(prism diopter)	Minimum, Maximum (Median)	0, 38 (15)	14, 49 (25)	<0.001	Mann–Whitney U-test
	95% Confidence interval	15.2 to 17.4	23.4 to 28.8		
Cyclotorsional deviation	Mean ± Standard deviation	−4.3 ± 5.4	−6.3 ± 5.5		
(degree)	Minimum, Maximum (Median)	−40, 4 (−3)	−21, 1 (−6.5)	0.02	Mann–Whitney U-test
-: excyclotorsion	95% Confidence interval	−3.6 to −5.1	−4.5 to −8.0		
+: incyclotorsion					
TNO stereoacuity					
15 to 60 sec of arc		60 (29.6%)	9 (20.9%)	0.09	Chi-square test
120 to 1980 sec of arc		62 (30.5%)	9 (20.9%)		
Not detected		81 (39.9%)	25 (58.1%)		
Dissociated vertical deviation					
Present		17 (8.4%)	1 (2.3%)	0.17	Fisher exact probability test
Absent		186 (91.6%)	42 (97.7%)		
Dominant eye					
Contralateral eye		120 (59.1%)	24 (55.8%)	0.69	Fisher exact probability test
Paretic eye		83 (40.9%)	19 (44.2%)		
Postoperative deviation					
Horizontal deviation	Mean ± Standard deviation	−2.6 ± 5.0	−3.3 ± 6.1		
(prism diopter)	Minimum, Maximum (Median)	−20, 12 (0)	−18, 8 (0)	0.37	Mann–Whitney U-test
	95% Confidence interval	−1.9 to −3.3	−1.4 to −5.2		
Vertical deviation	Mean ± Standard deviation	5.1 ± 5.3	7.0 ± 7.1		
(prism diopter)	Minimum, Maximum (Median)	0, 25 (3.5)	0, 35 (4.5)	0.04	Mann–Whitney U-test
	95% Confidence interval	4.4 to 5.9	4.7 to 9.3		

In the subgroup of patients with the idiopathic palsy (n = 212), the age at surgery was significantly older, vertical and cyclotorsional deviations were significantly larger in the repetitive-surgery group (n = 37), compared with the single-surgery group (n = 175) (*P* = 0.008, *P* < 0.001, *P* = 0.009, Mann–Whitney U-test, Table 4). The presence or the absence of dissociated vertical deviation did not show significant difference between the single-surgery group and the repetitive-surgery group (*P* = 0.16, Fisher exact probability test, Table 4). In the subgroup of patients with the acquired palsy (n = 34), vertical deviations only were significantly larger in the repetitive-surgery group (n = 6) than in the single-surgery group (n = 28) (*P* = 0.003, Table 4).

**Table 4:** **Clinical factors in the single-surgery group and repetitive-surgery group of either idiopathic or acquired superior oblique muscle palsy**

		**Idiopathic (n = 212)**			**Acquired (n = 34)**		
		**Single surgery**	**Repetitive surgeries**	***P*** **value**	**Single surgery**	**Repetitive surgeries**	***P*** **value**
		**(n = 175)**	**(n = 37)**		**(n = 28)**	**(n = 6)**	
Sex							
Male		86 (49.1%)	18 (48.6%)	0.96	21 (75.0%)	1 (16.7%)	0.25
Female		89 (50.9%)	19 (51.4%)		7 (25.0%)	5 (83.3%)	
Age at surgery	Mean ± Standard deviation	37.7 ± 26.5	49.2 ± 21.8		57.6 ± 12.1	61.8 ± 7.7	
(year)	Minimum, Maximum (Median)	3.4, 83.0 (39.7)	5.3, 82.8 (48.2)	0.008	36.5, 79.4 (56.8)	53.8, 76.6 (60.3)	0.25
	95% Confidence interval	33.8 to 41.7	41.9 to 56.4		53.0 to 62.4	53.8 to 70.0	
Horizontal deviation	Mean ± Standard deviation	−4.8 ± 8.4	−7.7 ± 13.9		−1.2 ± 7.1	−4.6 ± 16.3	
(prism diopter)	Minimum, Maximum (Median)	−35, 16 (−2)	−41, 17 (−6)	0.27	−20, 20 (0)	−37, 8 (0)	0.37
-: exodeviation,	95% Confidence interval	−3.6 to −6.1	−3.1 to −12.3		−4.0 to 1.6	−21.9 to 12.5	
+: esodeviation							
Vertical deviation	Mean ± Standard deviation	17.3 ± 7.7	26.9 ± 9.1		10.4 ± 7.9	21.5 ± 4.5	
(prism diopter)	Minimum, Maximum (Median)	2, 38 (16)	14, 49 (26)	<0.001	0, 32 (10)	15, 27 (22)	0.003
	95% Confidence interval	16.1 to 18.4	23.8 to 29.9		7.3 to 13.5	16.7 to 26.3	
Cyclotorsional deviation	Mean ± Standard deviation	−3.6 ± 4.3	−6.1 ± 5.5		−8.4 ± 8.6	−7.3 ± 6.3	
(degree)	Minimum, Maximum (Median)	−25, 4 (−3.0)	−21, 1 (−5.5)	0.009	−40, 1 (−7.5)	−16, 0 (−8.5)	0.45
-: excyclotorsion,	95% Confidence interval	−3.0 to −4.3	−4.2 to −8.0		−5.1 to −11.8	−0.7 to −14.0	
+: incyclotorsion							
TNO stereoacuity							
15 to 60 sec of arc		55 (31.4%)	7 (18.9%)		5 (17.9%)	2 (33.3%)	
120 to 1980 sec of arc		56 (32.0%)	9 (24.3%)	0.07	6 (21.4%)	0 (0%)	0.39
Not detected		64 (36.6%)	21 (56.8%)		17 (60.7%)	4 (66.7%)	
Dominant eye							
Contralateral eye		103 (58.9%)	20 (54.1%)	0.59	17 (60.7%)	4 (66.7%)	0.77
Paretic eye		72 (41.1%)	17 (45.9%)		11 (39.3%)	2 (33.3%)	
Dissociated vertical deviation							
Present		17 (9.7%)	1 (2.7%)	0.16	0 (0%)	0 (0%)	
Absent		158 (90.3%)	36 (97.3%)		28 (100%)	6 (100%)	

Statistical Methods are the same as stated in Table 3.

**Figure 1:**
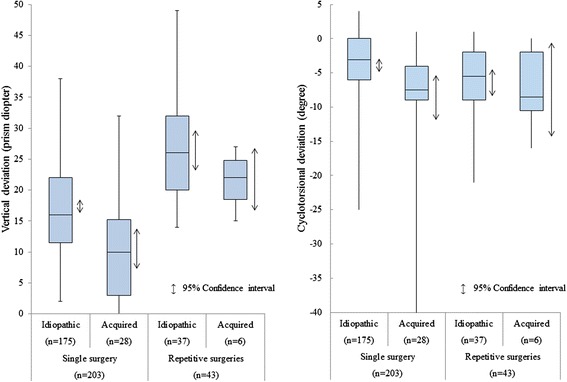
**Box plots for vertical deviations (left) and cyclotorsional deviations (right) in patients with idiopathic or acquired superior oblique muscle palsy who had a single surgery or repetitive (two or more) surgeries.** A box with a bar indicates the upper 75 percentile and lower 25 percentile, with a median, maximum, and minimum. Double-arrowhead bars indicate the 95% confidence interval. Note that the 95% confidence interval of vertical deviations is not overlapped with each other between the single-surgery group and repetitive-surgery group of idiopathic or acquired palsy. Minus degrees in cyclotorsional deviations indicate excyclotorsion.

The 95% confidence interval of vertical deviations in the single-surgery group and the repetitive-surgery group was not overlapped with each other in the overall analysis including the idiopathic and acquired palsy as well as in separate analyses for either the idiopathic palsy or the acquired palsy (Figure 1, Tables 3 and 4). Multivariate regression analysis was done in all 246 patients with either idiopathic or acquired superior oblique muscle palsy. The outcome of either a single surgery or repetitive surgeries was explained by three clinical factors; age at surgery (*P* = 0.3397), vertical deviation (*P* = 1.170 × e^−11^), and cyclotorsional deviation (*P* = 0.0369), indicating the vertical deviation as a key factor.

## Discussion

The goal of this study is to find clinical factors which give prediction for either a single surgery or two or more surgeries, required to treat successfully the idiopathic and acquired superior oblique muscle palsy. In the study period of 8 years, baseline surgical strategy for the idiopathic and acquired superior oblique muscle palsy at our institution was to plan a second surgery when the initial surgery was insufficient to treat diplopia or abnormal head posture. Basic surgical procedures, adopted as an initial surgery in this strategy, were inferior oblique muscle recession on the paretic side or inferior rectus muscle recession on the non-paretic side. The inferior oblique muscle recession on the paretic side was chosen basically when the non-paretic eye was the dominant eye, used for fixation. The inferior rectus muscle recession on the non-paretic side was chosen when the paretic eye was the dominant eye for fixation. A single surgical procedure was taken at a single secession of the surgery in most patients while two surgical procedures were done at a single secession only in the limited number of patients.

The preoperative factors with significant differences between the single-surgery group and the repetitive-surgery group, in the overall analysis of idiopathic and acquired superior oblique muscle palsy, were the age, vertical and cyclotorsional deviations. As expected from previous studies (Simons et al. 1998; Nejad et al. 2013; Morad et al. 2001), smaller vertical deviations and smaller cyclotorsional deviations resulted in successful alignment with a single secession of the surgery.

In the subgroup analysis of the idiopathic palsy, all three factors, the age, vertical and cyclotorsional deviations, remained significantly different between the single-surgery group and the repetitive-surgery group. In contrast, only the vertical deviation remained significantly different between the single-surgery and repetitive-surgery group of the acquired palsy. The low-grade torsion is concomitant in all directions of the gaze in the idiopathic palsy. In contrast, this is not true for the acquired palsy where the torsion is maximal in downward gaze in abduction. Therefore, the vertical deviation and the cyclotorsional deviation are interdependent on each other only in the idiopathic palsy. This fact will explain why cyclotorsional deviations were significantly larger in the group of idiopathic palsy with repetitive surgeries even though the torsion is typically low-grade in the idiopathic palsy.

It should be noted that torsion does not seem to be a factor predicting the need for repetitive surgery in the acquired palsy where torsion is generally large. This result might be attributed to the relatively small number of patients with the acquired palsy in this study. Under the circumstances, small vertical deviations were shown as a common predictive factor for a single surgery both in the idiopathic palsy and in the acquired palsy. The vertical deviation at the primary position of the gaze with the head kept straight, determined by the alternate prism and cover test, could be used as a common benchmark to predict the single surgery or the repetitive surgeries, required to treat both idiopathic and acquired superior oblique muscle palsy.

In the subgroup of patients with the idiopathic palsy, it is understandable that large vertical deviations are associated with the absence of stereopsis and poor stereoacuity at near fixation. The younger age, associated with a single surgery, in this group, is somewhat difficult to interpret. The younger patients with the idiopathic palsy might have more capability of gaining cyclovertical fusion, leading possibly to successful alignment with a single surgery.

The 95% confidence intervals of vertical deviations in the single-surgery group and the repetitive-surgery group did not overlap with each other in overall analysis, including both idiopathic and acquired superior oblique muscle palsy, and also in separate analysis for either idiopathic or acquired palsy. The vertical deviation can be measured by the alternate prism and cover test in standard ophthalmic clinics, and thus could be used as a common benchmark for predicting a single surgery or repetitive surgeries, required to treat either idiopathic or acquired superior oblique muscle palsy.

At closer look, however, outliers exist in the 95% confidence intervals of both the single-surgery group and the repetitive-surgery group: 32 (15.8%) of 203 patients with a single surgery (31 patients with idiopathic palsy and one with acquired palsy) showed vertical deviations of 24 prism diopters or greater while 5 (11.6%) of 43 patients with repetitive surgeries (4 patients with idiopathic palsy and one with acquired palsy) showed vertical deviations of 17 prism diopters or smaller. In the 32 patients with large vertical deviations in the single-surgery group, 21 patients showed cyclotorsional deviations of 5 degrees or smaller and 22 patients had stereopsis of 1980 seconds of arc or better. In the 5 patients with smaller vertical deviations in the repetitive-surgery group, only one patient showed a cyclotorsional deviation of 5 degrees or smaller and one patient had stereopsis. These facts suggest that a single surgery would be expected in patients with vertical deviations of 24 prism diopters or greater when they have small cyclotorsional deviations and better stereopsis.

The surgical strategy, described in this study, is to conduct a single surgical procedure first and to watch for a while, and then to plan a second procedure if additional correction is required. This stream of clinical decisions is easily followed by ophthalmologists who do not necessarily specialize in strabismus. The combination of two surgical procedures at a single secession of the surgery (Morad et al. 2001) is difficult to predict the outcome in general. In this context, lateral displacement of the rectus muscles, concurrent with the recession (von Noorden et al. 1996), would be also difficult to be followed, as a standard method, by general ophthalmologists. The preoperative assessment is important in choosing the best surgical approach in superior oblique muscle palsy. It is also mandatory to understand the difference between clinical features in the idiopathic palsy and the acquired palsy, such as the difference in vertical fusion amplitudes and the attitude of cyclotorsion.

## Conclusions

To our knowledge, this study presents the first scientific analysis to find clinical factors which underlie a single surgery or repetitive surgeries to treat successfully the idiopathic and acquired superior oblique muscle palsy. The analysis confirms a general view that small cyclovertical deviations can be corrected by a single surgery (Nejad et al. 2013). The 95% confidence interval of vertical deviations, at the primary position of the gaze with the head kept straight, would be used for a common benchmark for predicting a single surgery both for the idiopathic palsy and for the acquired palsy, in the process of obtaining the informed consent. The younger age is also found as another factor for predicting a single surgery only in the idiopathic palsy. On the other side of the coin, this study indicates that two-muscle surgery is indeed required for successful treatment in a minor group of patients with either idiopathic or acquired palsy.

## Methods

Retrospective review was made on medical records of 246 consecutive patients with idiopathic (n = 212) or acquired (n = 34) superior oblique muscle palsy who underwent surgeries in 8 years from January 2006 to December 2013 at Okayama University Hospital. The unilateral palsy was diagnosed in 236 patients and the bilateral palsy in 10 patients. Thirty seven patients with superior oblique muscle palsy in the 8-year period were excluded from this study because they had past history of strabismus surgery, or had the combined oculomotor or abducens palsy, or had mental or developmental delay. The study adhered to tenets of the Declaration of Helsinki and was approved as a retrospective study by the Ethics Committee of Okayama University Graduate School of Medicine, Dentistry, and Pharmaceutical Sciences.

The entity of idiopathic superior oblique muscle palsy included congenital and decompensated palsies. The decompensated palsy was diagnosed usually in adult patients who developed asthenopia or diplopia, and was confirmed by their pictures to have abnormal head postures since childhood. The acquired palsy included traumatic and ischemic palsies. The patients with acquired superior oblique muscle palsy were followed at least for half a year to wait the spontaneous recovery. Only the patients with symptoms, such as diplopia and asthenopia, underwent the surgery. Patients with combined palsies of abducens or oculomotor nerve were excluded from the study.

The 246 patients were divided into two groups, 203 patients with a single surgery only, and 43 patients with two or more surgeries to treat superior oblique muscle palsy: 36 patients with two surgeries, 6 with three surgeries, and one with four surgeries. The single-surgery group had 9 patients with the bilateral palsy (8 with the acquired palsy and one with the idiopathic palsy) while the repetitive-surgery group had one patient with the idiopathic bilateral palsy. Clinical factors, compared between the two groups, were the paretic or non-paretic eye as a dominant eye or an eye used for fixation, surgical methods, the sex, the age at initial surgery, horizontal, vertical, and cyclotorsional deviations, stereopsis at near fixation, and the presence or the absence of dissociated vertical deviation. In 10 patients with the bilateral palsy, the paretic eye was assigned to the eye with the more marked palsy.

The dominant eye was determined by asking a patient to peep through a hole of the card. The presence or the absence of stereopsis was determined and stereoacuity at near fixation was measured with TNO Stereotest (TNO test for stereoscopic vision, fifth edition, Lameris Ootech, Nieuwegein, The Netherlands). Horizontal and vertical deviations with the head kept straight were measured by alternate prism and cover test to the fixation light target at the distance of 5 m. Cyclotorsional deviations were measured with a major amblyoscope. Preoperative data were obtained within one month before the initial surgery while postoperative data were obtained around one month after the final surgery.

The initial surgical strategy in the study period, common in the idiopathic palsy and in the acquired palsy, was as follows: the inferior oblique muscle recession on the paretic side was chosen when the non-paretic eye was the dominant eye, used for fixation. In contrast, the inferior rectus muscle recession on the non-paretic side was chosen when the paretic eye was the dominant eye for fixation. In inferior oblique muscle recession, the anterior end and posterior end of the resected insertion were sutured at the points, 2 mm and 4 mm temporal, respectively, 5 mm posterior to the insertion of the lateral rectus muscle along its temporal border, based on the standard procedures (Parks 1972; Mims and Wood 1989; Ziffer et al. 1993). The length for recession of the rectus muscles was based on vertical and horizontal deviations, determined by the alternate prism and cover test.
